# Cellular Mechanisms Enabling Mitochondria Transfer and Transplantation

**DOI:** 10.1002/advs.78137

**Published:** 2026-10-06

**Authors:** F. Seyma Gokdemir, Yagmur Ozendi, Gokhan Burcin Kubat, Keshav K. Singh

**Affiliations:** ^1^ Institute of Food, Agriculture and Livestock Development Baskent University Ankara Türkiye; ^2^ Department of Food Processing, Kahramankazan Vocational School Baskent University Ankara Türkiye; ^3^ Institute of Transplantation and Gene Sciences Baskent University Ankara Türkiye; ^4^ Department of Exercise and Sport Sciences Faculty of Health Sciences Baskent University Ankara Türkiye; ^5^ Departments of Genetics, Dermatology and Pathology, Heersink School of Medicine University of Alabama at Birmingham Birmingham Alabama USA

**Keywords:** cell metabolism, inter‐organelle contact sites, mitochondria, mitochondrial DNA, mitochondrial transfer, mitochondrial transplantation, mitoTT

## Abstract

Mitochondrial transfer and transplantation (mitoTT) has emerged as an experimental and potentially transformative therapeutic strategy, drawing attention for its ability to modulate cellular bioenergetics, stress responses, and tissue repair. Endogenous mitochondrial transfer can occur through tunneling nanotubes, extracellular vesicles, cell fusion, gap junction‐associated communication, and phagocytosis‐like internalization. These routes differ substantially in distance, directionality, cargo integrity, regulatory control, and physiological relevance. Preclinical studies indicate that exogenous mitochondria may transiently improve bioenergetic function, attenuate oxidative stress, and support recovery in models of cardiac, skeletal muscle, and nervous system injury, whereas clinical evidence remains limited and heterogeneous. Broad clinical implementation remains constrained by unresolved biological and translational barriers. These include inefficient or poorly controlled uptake of extracellular mitochondria, uncertain long‐term persistence, incomplete functional integration, donor‐recipient incompatibility, mitochondrial‐nuclear mismatch, heteroplasmy‐related risks, immunogenicity, and the lack of standardized potency and delivery assays. Here, we critically synthesize the cellular mechanisms enabling mitoTT, emphasizing the checkpoints that determine whether transferred mitochondria are integrated, remodeled, immunologically sensed, or eliminated. We further discuss cristae biology and the mitochondrial contact site and cristae organizing system, mitochondrial nucleoid maintenance, mitochondrial DNA replication, organelle contact sites, and emerging bioengineering strategies required to move mitoTT from experimental rescue toward reproducible therapeutic application.

## Graphical abstract

Mitochondrial transfer and transplantation (mitoTT) is examined as a multistep process governed by distinct transfer routes and post‐transfer checkpoints. Mechanisms controlling mitochondrial uptake, network integration, cristae remodeling, mtDNA maintenance, immune sensing, and quality control are highlighted, revealing key biological and translational barriers that must be resolved to advance mitoTT toward safe, reproducible therapeutic application.

**Abbreviations**
ATP: Adenosine triphosphate
cGAS–STING: Cyclic GMP–AMP synthase–stimulator of interferon genes
DAMP: Damage‐associated molecular pattern
DRP1: Dynamin‐related protein 1
ECMO: Extracorporeal membrane oxygenation
ER: Endoplasmic reticulum
EV: Extracellular vesicle
FPR: Formyl peptide receptor
GMP: Good manufacturing practice
MAM: Mitochondria‐associated membrane
MAT: Mitochondrial augmentation therapy
MDV: Mitochondria‐derived vesicle
MICOS: Mitochondrial contact site and cristae organizing system
MIRO: Mitochondrial Rho GTPase
mito‐EV: Mitochondria‐containing extracellular vesicle
mitoTT: Mitochondrial transfer and transplantation
MQC: Mitochondrial quality control
MSC: Mesenchymal stromal/stem cell
mtDAMP: Mitochondrial damage‐associated molecular pattern
mtDNA: Mitochondrial DNA
NLRP3: NOD‐like receptor family pyrin domain‐containing 3
OPA1: Optic atrophy 1
OXPHOS: Oxidative phosphorylation
PGC‐1α: Peroxisome proliferator‐activated receptor gamma coactivator 1‐alpha
PINK1: PTEN‐induced kinase 1
POLG: DNA polymerase gamma
POLRMT: Mitochondrial RNA polymerase
ROS: Reactive oxygen species
TFAM: Mitochondrial transcription factor A
TLR9: Toll‐like receptor 9
TNT: Tunneling nanotube
TWNK: Twinkle mtDNA helicase

## 1 Introduction

Transfer of mitochondria between cells is recognized as a biological process with significant implications for tissue homeostasis and regeneration. Mitochondria were once thought to be strictly cell‐autonomous organelles but are now known to be shared between cells through several highly regulated mechanisms (Figure 1). Following transfer to a new cell, mitochondria can affect cellular metabolism, survival, and stress responses [1, 2, 3]. Mitochondrial transfer can rescue damaged cells, modulate immune signaling, and coordinate multicellular responses to damage. Such transfer events represent a dynamic form of intercellular communication.

**FIGURE 1:**
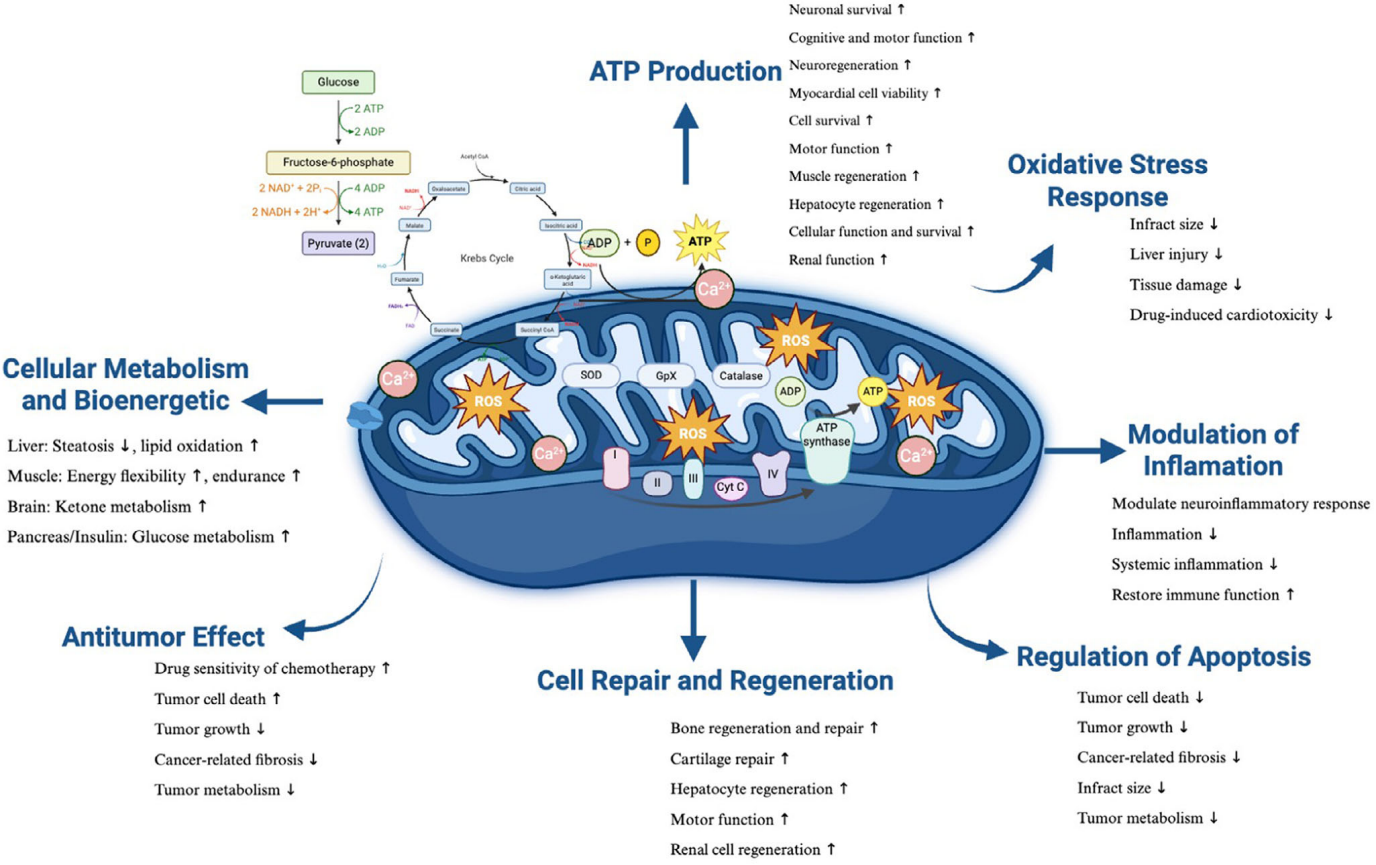
Schematic overview of mitochondrial metabolism and downstream cellular outcomes relevant to mitoTT. The figure summarizes how mitochondrial adenosine triphosphate (ATP) production, regulation of reactive oxygen species (ROS), and calcium signaling may influence bioenergetics, oxidative stress, inflammation, apoptosis, cell survival, and tissue repair. This schematic is intended as a conceptual overview of mitochondrial functional outputs rather than direct evidence that all listed outcomes occur after mitochondrial transfer in every cellular or disease context. The magnitude and durability of these effects depend on donor mitochondrial fitness, recipient‐cell stress status, uptake route, immune sensing, and post‐transfer quality control. Created with BioRender by F.S. Gokdemir (2026).

Several cellular routes have been implicated in mitochondrial transfer, but they should not be considered equivalent mechanisms because they differ in cargo type, spatial range, regulatory control, and the strength of experimental evidence supporting intact organelle transfer. Tunneling nanotubes (TNTs) are thin, actin‐rich membrane projections that support direct cell‐to‐cell trafficking of organelles and macromolecules, with mitochondrial movement regulated in part by actin‐based motors and mitochondrial trafficking adaptors such as mitochondrial Rho GTPase 1 (MIRO1) and, potentially, mitochondrial Rho GTPase 2 (MIRO2) [4, 5]. Extracellular vesicles (EVs), including exosomes, microvesicles, mitochondria‐derived vesicles (MDVs), and mitochondria‐containing extracellular vesicles (mito‐EVs), can transport mitochondrial DNA (mtDNA), mitochondrial proteins, respiratory components, or mitochondrial fragments; however, whether these vesicles routinely deliver fully intact and functional mitochondria remains context‐dependent [6]. In addition to mitochondrial cargo, EVs may carry immunomodulatory proteins and microRNAs (miRNAs), indicating that EV‐associated effects may reflect combined mitochondrial and non‐mitochondrial signaling [7]. Cell fusion enables cytoplasmic mixing and may permit large‐scale mitochondrial exchange in restricted biological contexts, whereas gap junction‐associated communication is better viewed as a mechanism for metabolic, ionic, and signaling coupling rather than a primary route for intact mitochondrial passage [8]. Finally, extracellular mitochondria can be internalized through phagocytosis‐like uptake, macropinocytosis, or receptor‐associated endocytic processes, all of which are particularly relevant to experimental mitoTT but also raise unresolved questions regarding endosomal escape, lysosomal degradation, and functional persistence [9, 10].

Once donor mitochondria or mitochondrial components enter recipient cells, their fate is determined by a sequence of intracellular checkpoints that govern retention, remodeling, functional integration, immune sensing, or elimination [11, 12]. Stable incorporation into the host mitochondrial network requires donor mitochondrial fitness, bioenergetic compatibility, and coordinated mitochondrial dynamics, including outer membrane fusion mediated by mitofusin 1 and 2 (MFN1/2), inner membrane remodeling regulated by optic atrophy 1 (OPA1), and fission‐dependent quality control involving dynamin‐related protein 1 (DRP1) [13]. Conversely, donor mitochondria that fail to maintain membrane potential, structural integrity, or compatibility with the recipient network may be eliminated through mitophagy, including PTEN‐induced putative kinase 1 (PINK1)‐Parkin‐dependent pathways [14]. In parallel, transferred mitochondria can become immunogenic when mitochondrial damage, failed uptake, lysosomal rupture, or cytosolic leakage exposes mitochondrial damage‐associated molecular patterns (mtDAMPs), including N‐formyl peptides and mtDNA, to formyl peptide receptors, Toll‐like receptor 9 (TLR9), the cyclic GMP‐AMP synthase‐stimulator of interferon genes (cGAS–STING) pathway, or inflammasome pathways [3, 15]. Functional persistence may also depend on whether transferred mitochondria re‐establish productive interactions with host organelles and organelle contact sites, particularly mitochondria‐associated endoplasmic reticulum membranes (MAMs), lysosomes, and peroxisomes, although direct evidence for stable post‐transfer organelle contact site reassembly remains limited [16].

Therapeutic mitoTT attempts to exploit these biological principles by delivering functional mitochondria or mitochondrial material to cells with impaired bioenergetic capacity [17]. Preclinical studies suggest that transplanted mitochondria may improve functional recovery in models of cardiac ischemia‐reperfusion injury, skeletal muscle damage, and neural injury; however, whether these effects reflect durable mitochondrial integration, transient metabolic support, paracrine signaling, or indirect immunomodulation often remains unresolved [18, 19, 20]. Thus, the central challenge for the field is to determine when mitochondrial transfer produces genuine organelle replacement, when it provides short‐lived bioenergetic rescue, and when it may generate maladaptive outcomes such as inflammation, tumor promotion, or mitochondrial genome incompatibility. In this review, we critically evaluate mitoTT as a multi‐step biological process, from transfer‐route selection and intracellular uptake to cristae remodeling, mitochondrial nucleoid maintenance, organelle contact site reassembly, immune sensing, quality control, and translational bioengineering.

## 2 The Mechanisms of Mitochondrial Transfer

### 2.1 Tunneling Nanotubes (TNTs)

TNTs are dynamic, actin‐rich membranous conduits that connect neighboring or spatially separated cells and support the directional trafficking of organelles, vesicles, ions, proteins, and signaling molecules, including mitochondrial cargo, over distances that can extend beyond direct membrane contact [4, 21] (Figure 2A). Mitochondrial morphology is likely to influence TNT‐mediated transfer because elongated mitochondria, fragmented mitochondria, or mitochondria‐derived vesicular material may differ in their ability to enter and move through narrow membranous conduits [22, 23]. Importantly, evidence for TNT‐associated mitochondrial exchange should be interpreted with attention to cargo identity: some studies support the transfer of intact mitochondria, whereas others suggest movement of mitochondrial fragments, mitochondria‐derived vesicles, mtDNA‐containing material, or signaling cargo rather than complete organelles [24].

**FIGURE 2:**
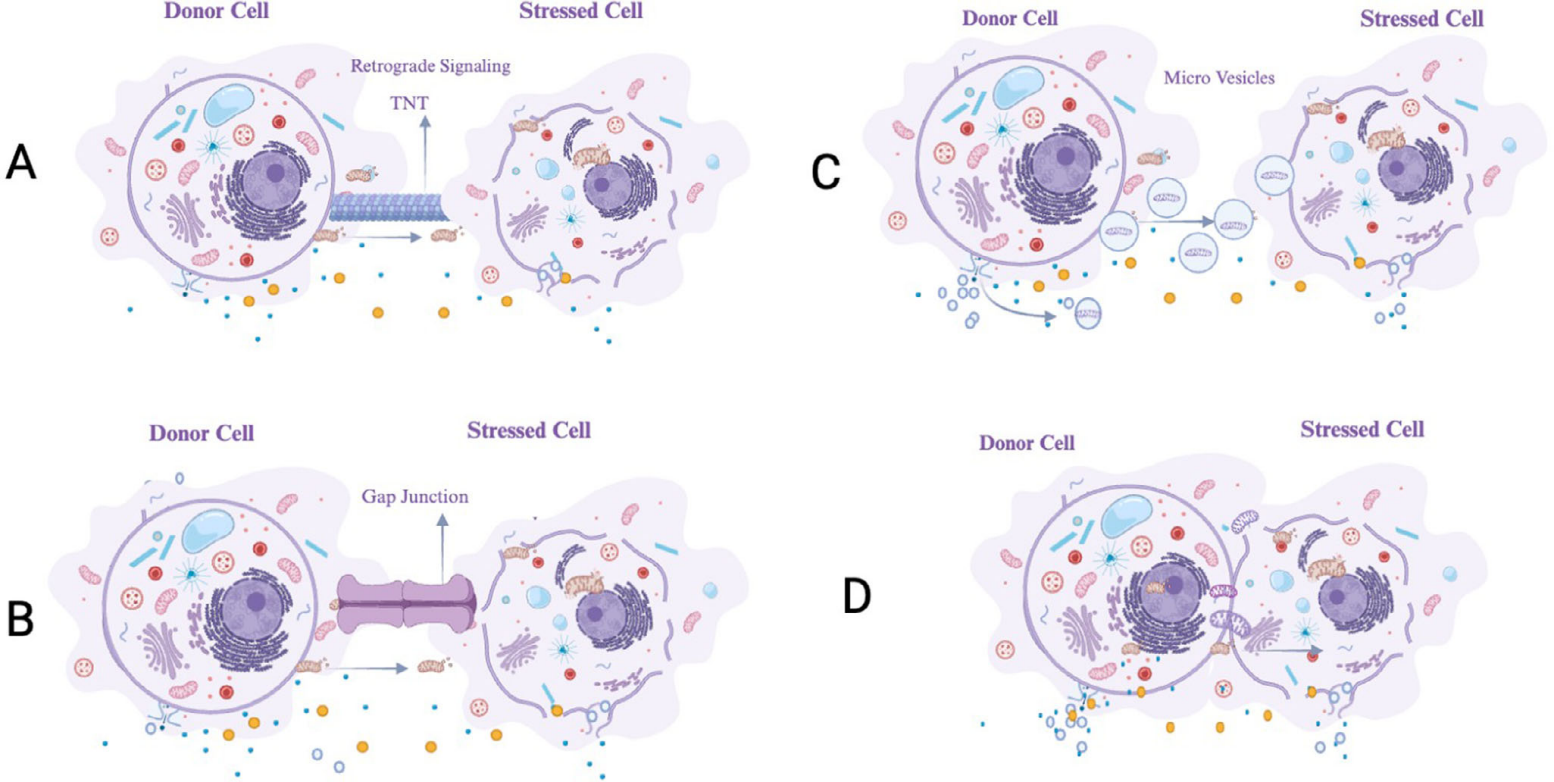
Major intercellular communication routes implicated in mitochondrial transfer and mitochondrial signaling. The schematic illustrates communication involving mitochondria or mitochondrial cargo between donor and stressed recipient cells through tunneling nanotubes (A), gap junction‐associated communication (B), microvesicle/extracellular vesicle release (C), and cell fusion (D). These mechanisms should not be interpreted as equivalent routes for intact mitochondrial transfer. TNTs and cell fusion may support direct organelle movement under specific cellular conditions, whereas extracellular vesicles may carry intact mitochondria, mitochondrial fragments, mitochondrial DNA (mtDNA), or mitochondrial proteins depending on vesicle subtype and disease context. Gap junctions are better understood as mediators of ionic, metabolic, and signaling coupling rather than primary conduits for intact mitochondrial passage. Adapted from [37] under the Creative Commons Attribution 4.0 International (CC BY 4.0) license. Created with BioRender by F. S. Gokdemir (2026).

TNT formation is commonly enhanced under cellular stress conditions, including oxidative stress, hypoxia, mitochondrial damage, calcium imbalance, inflammatory stimulation, and metabolic insufficiency. Mechanistically, TNT biogenesis depends on actin polymerization and membrane protrusion dynamics regulated by Rho‐family GTPases, cytoskeletal remodeling pathways, and stress‐responsive signaling networks such as phosphatidylinositol 3‐kinase (PI3K)‐protein kinase B (AKT)‐mechanistic target of rapamycin (mTOR) pathway, nuclear factor kappa B (NF‐κB) signaling, and p53‐associated pathways [25]. Mitochondrial trafficking through TNTs is coordinated by cytoskeletal motors and outer mitochondrial membrane adaptor proteins, particularly MIRO1, which links mitochondria to motor complexes and functions as a calcium‐sensitive regulator of mitochondrial motility. Although MIRO1 has received the greatest attention in TNT‐mediated mitochondrial transfer, MIRO2 may also contribute to mitochondrial positioning, motility, and donor–recipient trafficking efficiency; however, its specific role in TNT‐dependent intercellular transfer remains less well established [26]. The MIRO1‐trafficking kinesin‐binding protein (TRAK) axis has been implicated in mitochondrial positioning and movement along TNTs, thereby supporting donor‐to‐recipient mitochondrial trafficking and short‐term bioenergetic recovery under stress conditions [27, 28]. Consistent with this, TNT‐mediated mitochondrial exchange has been associated with improved recipient‐cell survival in models of energy deficit and tissue damage [28, 29].

Experimental disruption of actin polymerization using cytochalasin B, cytochalasin D, or latrunculin B reduces TNT formation and mitochondrial trafficking, supporting the actin dependence of TNT‐mediated transfer [30]. In contrast, microtubule disruption with nocodazole has a weaker or context‐dependent effect, suggesting that TNT formation is primarily actin‐driven, although microtubules may still contribute to cargo transport in specific cellular systems [31].

The biogenesis of TNTs is triggered under cellular stress conditions, including oxidative stress, mitochondrial damage, energy deficiency, or inflammatory stimuli [32]. p53 activation has also been implicated in TNT biogenesis, given its capacity to stimulate the formation of filopodia‐like protrusions on the plasma membrane. These protrusions can establish contacts with neighboring cells, contributing to TNT formation. Membrane fusion at these contact sites enables the formation of continuous cytoplasmic bridges. Cellular signaling pathways such as PI3K‐AKT‐mTOR and NF‐κB also control actin reorganization and trigger TNT formation [33, 34]. Phosphatidylserine (PS) externalization on stressed recipient cells may function as a “help‐me” signal that promotes directional interactions with donor cells, including mesenchymal stromal/stem cells (MSCs). Notably, PS externalization enhances TNT formation from MSCs toward damaged cells [35]. In several experimental systems, MSCs form TNT‐like connections with stressed epithelial, endothelial, immune, or neuronal cells and transfer mitochondrial cargo that can transiently improve ATP production, reduce oxidative stress, and enhance survival [36]. These findings suggest that TNT‐mediated transfer may contribute to the therapeutic effects observed after MSC administration in injured tissues, but the relative contribution of mitochondrial transfer compared with paracrine signaling, immunomodulation, and extracellular vesicle release remains difficult to quantify [29, 36]. Collectively, TNTs represent one of the better‐supported routes for direct intercellular mitochondrial exchange, particularly in stress‐responsive cell‐cell communication. However, cargo identity and functional persistence remain context‐dependent. In addition, their fragility, dependence on cell proximity, limited scalability, and uncertain contribution relative to other transfer routes remain important translational limitations.

### 2.2 Extracellular Vesicles and Mitochondrial Cargo Transfer

EVs, including exosomes and microvesicles, are membrane‐bound carriers that are released by cells that can mediate intercellular communication without requiring direct cell‐cell contact (Figure 2C). In the context of mitochondrial transfer, EVs may contain mtDNA, mitochondrial proteins, respiratory‐chain components, MDVs, mitochondrial fragments, or, in selected contexts, intact mitochondria [38, 39]. Encapsulation within a lipid bilayer may protect mitochondrial cargo from extracellular nucleases, proteases, calcium overload, and oxidative damage, but the functional competence of EV‐associated mitochondrial cargo depends on vesicle origin, isolation method, cargo integrity, and recipient‐cell uptake route. Exosomes are generated through endosomal multivesicular body pathways that may involve endosomal sorting complexes required for transport (ESCRT)‐dependent or ESCRT‐independent mechanisms, whereas microvesicles are produced by outward budding of the plasma membrane [40, 41]. Recipient cells can internalize EVs through clathrin‐mediated endocytosis, caveolin‐dependent uptake, macropinocytosis, phagocytosis‐like uptake, or membrane fusion, but these routes do not necessarily ensure cytosolic delivery or functional integration of mitochondrial cargo [41]. The transfer of EV‐associated mitochondrial cargo has been linked to metabolic reprogramming, redox regulation, and improved recipient‐cell survival in selected experimental systems. However, these effects may reflect a combination of mitochondrial cargo delivery, paracrine signaling, and immunomodulatory activity rather than direct mitochondrial replacement alone [39, 42]. Furthermore, the relative stability and engineering potential of EVs make them attractive candidates for systemic or targeted delivery, although biodistribution, targeting specificity, cargo heterogeneity, and uptake efficiency remain major limitations [39, 42]. The combination of advanced isolation methods, such as size‐exclusion chromatography and ultrafiltration, with surface ligand functionalization may improve therapeutic delivery and reduce immunogenicity, although these strategies still require standardized validation across disease models and clinical contexts [40, 42].

A key conceptual distinction should be made between EVs that carry mitochondrial signals and EVs that contain structurally preserved mitochondrial material. In this context, MDVs and mito‐EVs have been proposed as related but not identical categories of EV‐associated mitochondrial cargo [43]. MDVs are generally nanoscale vesicles, often reported to be approximately 70–150 nm in diameter, although their size and composition may vary depending on cell type, stress condition, and isolation method. MDVs can route selected mitochondrial components toward multivesicular bodies (MVBs), lysosomes, or secretory pathways through mechanisms involving sorting nexin 9 (SNX9), optic atrophy 1 (OPA1), and mitochondrial quality‐control machinery. Depending on vesicular routing, mitochondrial cargo such as mtDNA, translocase of the outer mitochondrial membrane 20 (TOM20), mitochondrial heat shock protein 70 (mtHSP70), respiratory‐chain proteins, or other mitochondrial components may be directed toward either lysosomal degradation or extracellular release through EV‐associated pathways [44, 45]. The Parkin‐Syntaxin‐17‐SNAP29‐VAMP7 axis links damaged mitochondrial cargo to autophagosomal or lysosomal routing. These processes are closely linked to mitochondrial quality control (MQC), but they do not necessarily ensure that only functional mitochondria or mitochondrial components are released through EV‐associated pathways [46].

Elevated calcium levels can disrupt mitochondrial membrane potential, promote permeability transition, and contribute to apoptosis or necrosis. The lipid bilayer of EV membranes may protect mitochondrial cargo from calcium overload, reactive oxygen species (ROS), and extracellular enzymatic damage [47]. Experimental studies suggest that EV‐associated mitochondrial cargo can preserve selected structural or bioenergetic features for limited periods under stress conditions such as elevated Ca^2^^+^ levels or exposure to hydrogen peroxide (H_2_O_2_) [3, 42, 48]. However, preservation of mitochondrial features within EVs does not necessarily demonstrate cytosolic release, network integration, or durable functional persistence after uptake by recipient cells.

EVs may provide a protective extracellular microenvironment that helps preserve selected mitochondrial cargo during intercellular transport. Unlike tunneling nanotubes or cell fusion, EV‐mediated communication does not require stable physical contact between donor and recipient cells [49]. This property may enable mitochondrial signals or cargo to reach spatially separated recipient cells through paracrine or systemic distribution routes [50]. Such contact‐independent communication may contribute to coordinated metabolic or inflammatory responses at the tissue level, although the extent of this coordination remains context‐dependent [51]. In contrast, direct transfer mechanisms such as TNTs and cell fusion depend on spatial proximity and structural continuity between cells, whereas EVs can circulate and be distributed more broadly through extracellular fluids [52]. However, this increased range may come at the cost of reduced targeting specificity, heterogeneous cargo composition, dilution in circulation, and uncertain uptake efficiency.

EV‐mediated transport, TNT‐dependent transfer, phagocytosis‐like uptake, and cell fusion should therefore be viewed as complementary rather than interchangeable processes, differing in range, efficiency, cargo integrity, directionality, and regulatory control [53, 54]. TNTs provide contact‐dependent and potentially directional transfer, whereas EVs enable contact‐independent dissemination of mitochondrial signals or cargo with greater scalability but less predictable targeting.

### 2.3 Cell Fusion

Cell fusion is the process by which the plasma membranes of two adjacent cells merge, generating a shared cytoplasmic compartment that can permit exchange of organelles, metabolites, and signaling molecules (Figure 2D). As a result, mitochondria exchange may occur on a larger scale than through vesicular or nanotube‐mediated transfer, although this exchange occurs together with broader cytoplasmic and sometimes nuclear mixing. Physiological cell fusion occurs in defined contexts such as muscle development, trophoblast differentiation, and selected stem‐cell niche interactions [55, 56]. This process is regulated by context‐specific fusogenic proteins, including syncytins, dendritic cell‐specific transmembrane protein (DC‐STAMP), and myomaker, together with membrane‐remodeling and actin‐cytoskeletal pathways that enable membrane apposition and fusion [56]. Following fusion, donor‐derived mitochondria may become part of the shared mitochondrial population and contribute to ATP production, redox regulation, and metabolic remodeling, although the extent of stable integration depends on compatibility with the recipient‐cell environment [55]. Such mitochondrial sharing may be particularly relevant in cell types with high energy demands or high stress sensitivity, including cardiomyocytes and MSCs [57, 58]. For example, experimental studies suggest that interactions between MSCs and cardiomyocytes in ischemic or injured cardiac settings can support oxidative phosphorylation and reduce oxidative stress, although the relative contributions of complete cell fusion, TNTs, EVs, and paracrine signaling may be difficult to distinguish. More broadly, fusion‐like interactions involving endogenous progenitor cells, transplanted MSCs, or pluripotent stem cell‐derived cells may provide metabolic support during tissue repair, but the evidence varies across in vitro co‐culture systems, animal injury models, and disease contexts [57, 59].

Recent studies suggest that cell fusion can act as an adaptive response in selected pathological conditions associated with mitochondrial dysfunction, severe energetic stress, or tissue injury. In damaged or stressed tissues, donor cells such as MSCs or endogenous progenitor‐like cells may fuse with metabolically impaired cells and transfer functional mitochondria or mitochondrial networks, thereby supporting bioenergetic recovery and cell survival in specific experimental settings [27, 60]. In contrast to regenerative settings, cell fusion‐mediated mitochondrial exchange in the tumor microenvironment may support maladaptive outcomes. Cancer cells may fuse with stromal, immune, or other tumor‐associated cells, enabling exchange of mitochondria and metabolic components that can enhance metabolic plasticity, survival under stress, metastatic potential, and therapy resistance [11, 61]. Therefore, in cancer, mitochondrial exchange through fusion should be interpreted not as regenerative rescue but as a potential mechanism of metabolic adaptation and treatment escape.

Mitochondrial transfer through cell fusion may also influence cellular reprogramming, differentiation, and cell‐fate decisions. Introduction of donor mitochondrial content can alter intracellular signaling, redox status, metabolite availability, and mitochondrial‐nuclear crosstalk, thereby potentially influencing epigenetic regulation and cell identity [62, 63]. Thus, mitochondrial exchange via cell fusion represents not only a structural consequence of cytoplasmic mixing but also a potential regulator of cellular fate. Cell fusion is a potentially high‐capacity but context‐dependent mechanism of mitochondrial exchange. Although direct cytoplasmic continuity may enable rapid metabolic support, cell fusion is largely restricted to specific biological settings, including development, tissue repair, immune interactions, and tumor microenvironments. Compared with TNTs and EVs, cell fusion may permit larger‐scale mitochondrial exchange but is less spatially selective, less readily controlled, and more difficult to harness therapeutically.

### 2.4 Gap Junctions

Gap junctions are intercellular channels that permit the direct exchange of ions, second messengers, and small metabolites between adjacent cells, thereby supporting electrical and metabolic coupling in tissues such as the heart (Figure 2B) [64]. These channels are formed by connexin proteins, which assemble into hemichannels on neighboring cells and dock to create aqueous pores that allow passage of ions and small signaling molecules. In the context of mitochondrial transfer, gap junctions are best interpreted as modulators of mitochondrial signaling and homeostasis rather than as primary conduits for intact mitochondrial movement [52, 65]. Connexin channel assembly, permeability, and turnover are dynamically regulated by phosphorylation and other post‐translational modifications downstream of kinase signaling pathways [66]. The PI3K‐AKT pathway can influence cardiomyocyte growth, survival, metabolism, and connexin 43 (Cx43)‐dependent intercellular communication. In pluripotent stem cell‐derived MSCs, Cx43‐dependent gap junctional communication has been linked to enhanced mitochondrial transfer capacity, suggesting that connexin‐mediated coupling may facilitate donor–recipient coordination during mitochondrial exchange rather than physically transport intact mitochondria through the channel itself [67, 68]. Cx43 is an integral membrane protein synthesized in the endoplasmic reticulum and oligomerized in the Golgi apparatus to form connexons, or hemichannels [69]. Hemichannels on adjacent cells dock with one another to generate functional gap junction channels. Through these channels, cells exchange ions, metabolites, and small signaling molecules that can indirectly influence mitochondrial membrane potential, calcium handling, redox balance, and metabolic state [70, 71]. Connexins, particularly Cx43, have also been detected in mitochondrial compartments, where they have been implicated in the regulation of potassium flux, respiration, mitochondrial permeability transition, and ROS production [69, 72]. In ischemic or injured tissues, Cx43‐mediated communication may help preserve ionic homeostasis, metabolic coupling, and stress‐response coordination, thereby indirectly supporting mitochondrial function and cellular survival [73, 74]. Consequently, gap junctions should be considered complementary regulators of intercellular metabolic coordination rather than independent high‐capacity routes for mitoTT. Their contribution to mitochondrial transfer is therefore likely to be context‐dependent, involving support of donor‐recipient communication, calcium and redox coupling, and tissue‐level stress adaptation rather than direct passage of intact mitochondria [75].

### 2.5 Phagocytosis‐Like Internalization and Endocytic Uptake of Extracellular Mitochondria

Phagocytosis‐like internalization refers to an endocytic process through which cells internalize extracellular particles, vesicles, organelles, or organelle‐derived material, including mitochondria, under selected experimental conditions [76]. This mechanism is particularly relevant to mitoTT because exogenous mitochondria delivered into extracellular spaces must first bind to the recipient‐cell surface and then enter the cell through endocytic or phagocytosis‐like routes before any functional effect can occur [60]. Although this process shares features with classical phagocytosis, mitochondrial uptake is not necessarily equivalent to pathogen clearance; rather, it may support metabolic adaptation, stress recovery, or mitochondrial quality‐control responses depending on cellular context. Therefore, phagocytosis‐like mitochondrial uptake can be interpreted as a context‐dependent interface between cellular defense pathways, membrane trafficking, and bioenergetic regulation [60, 76]. Mitochondrial uptake is accompanied by cytoskeletal remodeling, particularly actin filament rearrangement, with actin polymerization driving membrane protrusions that surround or engulf extracellular mitochondria [77]. Such protrusions may resemble lamellipodia or filopodia and facilitate mitochondrial engulfment and internalization [78].

This uptake process is regulated by signaling and trafficking proteins, including PI3K, Rac family small GTPase 1 (Rac1), p21‐activated kinase 1 (PAK1), and dynamin [79]. Consistent with this mechanism, inhibition of actin polymerization by cytochalasin D or latrunculin B reduces mitochondrial uptake, supporting the actin‐dependent nature of this process [77]. Internalization may occur through macropinocytosis or related endocytic routes, in which membrane ruffles or invaginations generate vesicular compartments containing extracellular mitochondria. A critical unresolved question is whether internalized mitochondria escape from endosomal compartments into the cytosol, fuse with the host mitochondrial network, or instead undergo lysosomal degradation [80]. Under conditions of energy deficiency, oxidative stress, or membrane damage, recipient cells may display or upregulate recognition‐associated molecules such as integrin β1, CD44, translocase of the outer mitochondrial membrane 20 (TOM20), and PS, which may facilitate binding or uptake of extracellular mitochondria [81, 82]. These recognition events may couple extracellular mitochondrial binding to actin‐dependent internalization. PS externalization may act as a “help‐me” signal that promotes interactions between stressed recipient cells and mitochondria donor cells, including MSCs [83, 84]. Through this mechanism, damaged cells may acquire exogenous mitochondrial material that supports partial bioenergetic recovery, although the durability and integration status of the acquired mitochondria remain context‐dependent [85]. Neurons, cardiomyocytes, and endothelial cells have been reported to internalize extracellular mitochondria through phagocytosis‐like or macropinocytic mechanisms, with functional improvements observed in selected experimental models [86, 87]. Reported downstream effects include increased ATP production, reduced ROS accumulation, improved mitochondrial respiration, and decreased apoptosis, but these outcomes should be interpreted together with evidence for mitochondrial persistence, localization, and network integration [88]. These observations support phagocytosis‐like internalization as a plausible uptake route for exogenous mitochondria, but the efficiency of this route varies across recipient‐cell types and experimental conditions [87, 89]. Overall, phagocytosis‐like internalization represents a major candidate mechanism for cellular uptake of extracellular mitochondria, particularly in experimental mitoTT. However, its therapeutic relevance depends not only on uptake efficiency but also on endosomal escape, avoidance of lysosomal degradation, maintenance of mitochondrial membrane potential, immune tolerance, and durable functional integration.

### 2.6 Comparative Interpretation of Mitochondrial Transfer Routes

The mitochondrial transfer routes described above should be interpreted as complementary but mechanistically distinct processes rather than interchangeable pathways. TNTs and cell fusion provide direct physical continuity between donor and recipient cells, whereas EV‐mediated transfer and phagocytosis‐like uptake can occur without stable donor–recipient contact. Gap junctions, in contrast, are better understood as supportive mediators of ionic, metabolic, and signaling coordination rather than primary conduits for intact mitochondrial passage.

In acute injury settings, such as ischemia, oxidative damage, or trauma, mitochondrial transfer may function primarily as a rapid stress‐response mechanism that provides transient bioenergetic support, redox buffering, or immunomodulatory signaling. In these contexts, TNT‐mediated transfer, MSC‐associated mitochondrial donation, EV‐mediated mitochondrial cargo delivery, and uptake of extracellular mitochondria may all contribute to short‐term cellular recovery, although their relative contributions are often difficult to separate experimentally. In chronic disease settings, including neurodegeneration, cancer, metabolic dysfunction, or persistent inflammation, the same transfer mechanisms may have more complex consequences. Transferred mitochondria may support adaptation and survival of damaged cells, but they may also promote maladaptive outcomes such as inflammatory amplification, tumor metabolic plasticity, therapy resistance, or persistence of incompatible mitochondrial genomes.

For this reason, therapeutic relevance should not be assigned solely based on whether mitochondrial transfer has been observed. A stronger therapeutic relevance rating should require reproducible evidence of mitochondrial uptake, preservation of mitochondrial membrane potential or respiratory activity, measurable improvement in recipient‐cell function, and in vivo efficacy, together with biodistribution or persistence data and a disease‐context‐specific safety assessment. Conversely, mechanisms supported mainly by in vitro observations, indirect mitochondrial markers, or short‐term functional rescue should be considered promising but incompletely validated. Table 1 summarizes these routes according to cargo type, biological range, directionality, validation status, advantages, limitations, and therapeutic interpretability.

**TABLE 1:** Comparative features, evidence strength, and therapeutic interpretability of mitochondrial transfer routes.

Mechanism	Main cargo / range	Contact requirement / directionality	Evidence strength and representative contexts	Advantages	Key limitations and therapeutic interpretability
TNTs	Intact mitochondria, mitochondrial fragments, vesicular mitochondrial material, or signaling cargo; short‐to‐intermediate cell‐to‐cell range	Contact‐dependent; potentially directional	Strong evidence for direct transfer in selected models; reported in MSCs, neurons, immune cells, epithelial and stromal cells	Directional organelle trafficking; rapid stress‐responsive support	Fragile structures; requires proximity; difficult to scale therapeutically; cargo identity can vary. Highly promising for local tissue repair models but limited for systemic delivery [4, 21, 25, 28]
EVs / mito‐EVs / MDVs	mtDNA, mitochondrial proteins, respiratory components, mitochondrial fragments, and occasionally intact mitochondria; paracrine to systemic range	Contact‐independent; targeting less predictable	Moderate evidence; strong for mitochondrial cargo, variable for intact functional mitochondria; reported in MSCs, immune cells, endothelial cells, tumor cells, and neural cells	Stable carrier platform; bioengineerable; potential systemic delivery	Cargo heterogeneity; isolation artifacts; biodistribution uncertainty; uptake does not prove integration. Promising but incompletely validated [38, 39, 42, 43]
Cell fusion	Large‐scale cytoplasmic and organelle mixing; intact mitochondria possible; adjacent cells/tissue‐local range	Contact‐dependent; non‐selective cytoplasmic continuity	Moderate and context‐dependent evidence; reported in muscle, trophoblasts, MSC–cardiomyocyte systems, and tumor microenvironments	High‐capacity mitochondrial exchange	Less controlled; may involve nuclear/cytoplasmic mixing; restricted biological contexts; tumor‐promoting risk. Context‐dependent, potentially useful in repair but risky in cancer [53, 57, 59, 60]
Gap junction‐associated communication	Ions, metabolites, and second messengers; indirect mitochondrial signaling; adjacent‐cell range	Contact‐dependent; small‐molecule coupling	Moderate evidence for metabolic coupling but limited evidence for intact mitochondrial transfer; reported in cardiomyocytes, neural cells, and stem/stromal cells	Supports calcium, redox, and metabolic coordination; Cx43‐linked communication	Not a primary route for intact mitochondrial passage; mainly indirect. Supportive rather than a standalone mitoTT route [68, 69, 70, 72]
Phagocytosis‐like uptake / macropinocytosis	Extracellular mitochondria, mitochondrial particles, or organelle‐derived material; local extracellular uptake	Contact‐independent after extracellular delivery; recipient‐driven uptake	Moderate to strong evidence for uptake, variable evidence for durable integration; reported in neurons, cardiomyocytes, endothelial cells, immune and stromal cells	Directly relevant to exogenous mitoTT; broad recipient‐cell applicability	Endosomal trapping; lysosomal degradation; uncertain cytosolic escape; risk of immune sensing. Promising for transplantation but dependent on persistence and integration [9, 10, 60, 87, 89]

**Abbreviations**: Cx43, connexin 43; EVs, extracellular vesicles; MDVs, mitochondria‐derived vesicles; mito‐EVs, mitochondria‐containing extracellular vesicles; mitoTT, mitochondrial transfer and transplantation; MSCs, mesenchymal stromal/stem cells; mtDNA, mitochondrial DNA; TNTs, tunneling nanotubes.

## 3 Post‐Transfer Fate of Donor Mitochondria: Integration, Remodeling, Immune Sensing, or Elimination

After mitoTT, the biological outcome is not determined solely by cellular uptake. Donor mitochondria must retain membrane potential, avoid premature degradation, interact with the recipient cell's intracellular environment, and either integrate into the endogenous mitochondrial network or be eliminated through quality‐control pathways. Therefore, the post‐transfer fate of donor mitochondria can be conceptualized as a sequence of checkpoints involving mitochondrial dynamics, cristae architecture, nucleoid maintenance, mtDNA replication, organelle contact‐site reassembly, innate immune sensing, and mitophagic clearance. These checkpoints determine whether transferred mitochondria achieve durable functional integration, provide transient bioenergetic support, trigger inflammatory activation, or undergo degradation.

### 3.1 Network Integration: Fusion, Fission, and Mitophagic Selection

MitoTT aims to restore bioenergetic capacity in cells affected by mitochondrial dysfunction, but therapeutic benefit requires more than the physical entry of donor mitochondria into recipient cells [3, 63, 89]. After uptake, donor mitochondria must either fuse with the endogenous mitochondrial network, persist as functionally independent organelles, or be selectively removed if they are damaged, depolarized, or incompatible with the host cellular environment [90]. Transferred mitochondria delivered through endocytic uptake, TNTs, EV‐associated routes, or cell fusion may require coordinated interaction with the recipient mitochondrial network to achieve durable functional integration. Outer mitochondrial membrane fusion is mediated by MFN1/2, which can support structural continuity between donor and host mitochondrial populations when donor mitochondria are sufficiently viable and compatible [91]. In addition, MFN2 regulates mitochondria‐endoplasmic reticulum contact sites, which may influence calcium homeostasis, lipid exchange, and intracellular signaling after transfer [92]. Some experimental models report increased MFN1/2 expression after mitoTT, together with improved oxidative phosphorylation and ATP production; however, increased fusion‐marker expression alone does not prove stable donor–host mitochondrial network integration [93].

Mitochondrial fission is regulated by dynamin‐related protein 1 (DRP1), which promotes mitochondrial division and can facilitate both network remodeling and segregation of damaged mitochondrial segments. In the post‐transfer setting, fission may help segregate damaged donor‐derived material, remodel mitochondrial morphology, or generate mitochondrial fragments that are either reintegrated or targeted for degradation [62]. Donor mitochondria that fail to maintain membrane potential, respiratory competence, or network compatibility may be targeted for mitophagy through DRP1‐associated and PINK1–Parkin‐dependent quality‐control pathways [63, 94]. In cardiac ischemia‐reperfusion models, modulation of mitochondrial dynamics has been associated with improved mitochondrial stability and function, although whether these effects reflect direct donor mitochondrial integration or broader remodeling of the host network remains unresolved [95].

The balance between mitochondrial fusion and fission is critical for maintaining bioenergetic stability. Excessive fusion may propagate damaged mitochondrial components within the network, whereas excessive fission can promote fragmentation, respiratory impairment, and mitophagic clearance [96]. Therefore, the tightly regulated activities of MFN1/2 and DRP1 are essential for sustaining mitochondrial function after mitoTT. Pharmacological modulation of mitochondrial dynamics has been proposed as a potential means of creating a more permissive environment for post‐transfer mitochondrial persistence. However, direct evidence that MFN2 activation or DRP1 inhibition improves durable donor mitochondrial integration after mitoTT remains limited [93]. In neurodegenerative and cardiac diseases, impaired MFN‐mediated fusion and excessive DRP1 activity are associated with mitochondrial dysfunction, fragmentation, and reduced bioenergetic capacity [97, 98]. In these contexts, mitophagy acts as a key selection mechanism by eliminating damaged, depolarized, or non‐integrated mitochondria, thereby preserving cellular homeostasis and shaping the persistence of donor‐derived mitochondrial material [99]. Thus, post‐transfer success depends on a regulated balance between fusion, fission, and mitophagic selection, but changes in these pathways should be interpreted alongside direct evidence of donor mitochondrial persistence, localization, respiratory function, and compatibility with the host network.

### 3.2 Cristae Remodeling and MICOS‐Dependent Ultrastructural Adaptation

Functional integration of transferred mitochondria requires not only outer membrane fusion or cytoplasmic persistence but also preservation or remodeling of inner mitochondrial membrane architecture. Cristae are invaginations of the inner mitochondrial membrane that organize respiratory‐chain complexes, shape local proton gradients, influence calcium handling, and regulate susceptibility to cytochrome c release and apoptosis [100, 101, 102]. Therefore, donor mitochondria that are internalized but fail to maintain cristae organization may show limited oxidative phosphorylation, increased ROS production, or enhanced sensitivity to mitophagic clearance. This distinction is important because improved ATP production after mitoTT does not necessarily prove durable ultrastructural integration of donor mitochondria into the host network.

The mitochondrial contact site and cristae organizing system (MICOS) complex is a central scaffold of cristae junction architecture. MICOS is commonly described as a multi‐subunit complex containing MIC60, MIC10, MIC19, MIC25, MIC13, MIC26, and MIC27, with MIC60‐ and MIC10‐containing subcomplexes contributing to cristae junction formation, inner membrane curvature, and contact with outer membrane organizing machineries [103, 104, 105, 106]. In parallel, optic atrophy 1 (OPA1), cardiolipin, and F1Fo‐ATP synthase dimerization or oligomerization cooperate with MICOS to maintain cristae junction stability and respiratory‐chain organization [100, 102, 103]. In the context of mitoTT, these mechanisms are likely factors that determine donor mitochondrial respiratory competence and cristae adaptability, but it is not yet clear how directly they contribute to post‐transfer remodeling.

Direct evidence that transplanted mitochondria re‐establish normal cristae architecture after uptake remains limited. Many studies assess donor mitochondrial function through ATP production, oxygen consumption, membrane potential, or recipient‐cell survival, but fewer directly examine cristae density, cristae junction width, MICOS integrity, respiratory supercomplex organization, or donor‐specific ultrastructural persistence. Future mitoTT studies should therefore combine functional assays with ultrastructural and molecular readouts, including transmission electron microscopy, electron tomography, super‐resolution imaging of MICOS/OPA1 components, respiratory supercomplex analysis, and donor‐specific mitochondrial tracking. Such analyses would help distinguish true organelle integration from short‐term metabolic rescue mediated by partial cargo transfer, paracrine signaling, or transient mitochondrial persistence.

### 3.3 Mitochondrial Nucleoid Maintenance and mtDNA Replication after Transfer

Another unresolved determinant of post‐transfer mitochondrial fate is whether donor mitochondria preserve their mtDNA nucleoids and maintain replication competence after entering recipient cells. Mammalian mtDNA is packaged into nucleoprotein structures known as mitochondrial nucleoids, which organize mtDNA replication, transcription, repair, and inheritance within the mitochondrial network [107, 108, 109]. These nucleoids are not passive DNA aggregates; rather, they are dynamic regulatory units that link mitochondrial genome maintenance to respiratory‐chain biogenesis and cellular metabolic demand.

Mitochondrial transcription factor A (TFAM) is a central mtDNA‐packaging protein that stabilizes mtDNA and regulates nucleoid architecture, whereas DNA polymerase gamma (POLG), Twinkle helicase (TWNK), mitochondrial single‐stranded DNA‐binding protein 1 (SSBP1), and mitochondrial RNA polymerase (POLRMT) coordinate mtDNA replication and transcription [110, 111, 112]. Therefore, transferred mitochondria that retain membrane potential and respiratory activity may still fail to persist if nucleoid integrity, mtDNA copy number, or replication machinery is compromised. On the other hand, if donor mtDNA is preserved, replicated, and transcriptionally regulated within the recipient‐cell environment, maintenance of donor nucleoids should promote longer‐term bioenergetic competence. This possibility should be explicitly tested in mitoTT studies.

This issue is especially important for mitoTT because functional improvement after mitochondrial uptake does not necessarily demonstrate donor mtDNA persistence. Short‐term increases in ATP production or oxygen consumption may reflect transient activity of imported organelles, partial mitochondrial cargo transfer, or indirect signaling rather than stable maintenance of donor mitochondrial genomes. Long‐term integration would require evidence that donor mtDNA persists over time, avoids degradation, maintains appropriate copy number, and remains compatible with recipient nuclear‐encoded mitochondrial proteins.

Donor–recipient compatibility also raises the issue of mitochondria‐nuclear coordination and heteroplasmy. Because oxidative phosphorylation depends on coordinated expression of mtDNA‐encoded and nuclear‐encoded subunits, donor mtDNA that is poorly matched to the recipient nuclear background may impair respiratory‐chain assembly, increase ROS production, or trigger selective elimination through mitochondrial quality control pathways [113, 114, 115]. In allogeneic or cross‐lineage mitoTT, this risk may be more relevant than in autologous mitoTT, although the degree to which donor mtDNA persists and contributes to heteroplasmic states remains insufficiently characterized.

Future mitoTT studies should therefore include donor‐specific mtDNA tracking in addition to functional assays. Useful readouts may include donor–recipient mtDNA variant tracing, mtDNA copy number analysis, single‐cell or digital polymerase chain reaction (PCR)‐based heteroplasmy assessment, TFAM and nucleoid imaging, long‐read or deep sequencing to assess donor mtDNA persistence, and parallel measurement of respiratory‐chain function. These approaches would help distinguish transient metabolic rescue from durable mitochondrial genome maintenance and would clarify whether transferred mitochondria are functionally integrated, genetically maintained, or progressively eliminated.

### 3.4 Mitophagic Degradation and Immune Sensing of Transferred Mitochondria

The PINK1‐Parkin pathway plays a central role in mitochondrial quality control and is likely to influence the persistence or elimination of transferred mitochondria after uptake. Under normal conditions, PINK1 is rapidly degraded in polarized mitochondria; upon mitochondrial damage and membrane depolarization, PINK1 accumulates on the outer mitochondrial membrane, marking mitochondria for mitophagic clearance [116]. PINK1 accumulation recruits the E3 ubiquitin ligase Parkin from the cytosol, leading to ubiquitination of outer mitochondrial membrane proteins. These ubiquitin marks promote recognition by the autophagy adaptors and delivery of damaged mitochondria to the autophagosome–lysosome system [117]. Ubiquitinated outer membrane proteins are recognized by autophagy machinery through adaptor proteins such as p62/ Sequestosome 1 (SQSTM1) and Microtubule‐Associated Protein 1 Light Chain 3 (LC3)‐associated pathways [118]. Consequently, damaged or depolarized mitochondria are engulfed by autophagosomes and ultimately delivered to lysosomes for degradation. In the post‐transfer setting, PINK1–Parkin‐dependent mitophagy may remove donor mitochondria that fail to maintain membrane potential, respiratory competence, or compatibility with the host mitochondrial network [95, 119]. However, PINK1–Parkin signaling should not be interpreted solely as a barrier to mitoTT; it may also protect recipient cells by preventing persistence of damaged or incompatible donor mitochondria [120]. Pharmacological modulation of mitophagy may therefore need to be carefully balanced, because excessive mitophagy could eliminate therapeutically useful donor mitochondria, whereas insufficient mitophagy could permit persistence of dysfunctional or immunogenic mitochondrial material.

Because of their bacterial ancestry, mitochondria contain N‐formylated peptides and other molecular patterns that are normally compartmentalized within intact organelles [121]. During mitochondrial damage, failed uptake, extracellular degradation, or lysosomal rupture after mitoTT, these molecules may become exposed to innate immune sensors, including formyl peptide receptors 1 and 2 (FPR1/2). FPR activation can trigger chemotaxis, oxidative burst responses, and cytokine release, thereby converting mitochondrial material into a damage‐associated inflammatory signal [15, 122]. Thus, immune pathways originally optimized to detect microbial or damage‐associated signals may also respond to damaged or poorly controlled transplanted mitochondria, potentially amplifying tissue inflammation [123].

mtDNA can function as a mitochondrial damage‐associated molecular pattern (mtDAMP) because of its bacterial‐like features, including a circular structure and unmethylated CpG motifs [124]. When mtDNA is released extracellularly, retained in damaged endolysosomal compartments, or leaked into the cytosol, it can activate endosomal Toll‐like receptor 9 (TLR9), cyclic GMP–AMP synthase–stimulator of interferon genes (cGAS–STING), and the (NLR Family Pyrin Domain Containing 3) NLRP3 inflammasome, leading to type I interferon responses and proinflammatory cytokine production [124]. This response may contribute to the recognition of transplanted mitochondria as foreign‐like or damage‐associated material, particularly when mitochondrial isolation, delivery, or intracellular routing compromises membrane integrity. Importantly, immune activation after mitoTT is likely to be context‐dependent: in acute injury, limited innate immune activation may participate in debris clearance and repair signaling, whereas in chronic neurodegenerative, inflammatory, or metabolic disease settings, persistent mtDAMP exposure may worsen tissue dysfunction. Similarly, autologous mitoTT is expected to reduce alloimmune recognition and donor–recipient mismatch, whereas allogeneic or cross‐lineage mitochondrial sources may increase the need for compatibility testing and inflammatory monitoring.

Clinically, mitigation of immune activation during mitoTT requires strategies that preserve mitochondrial integrity and limit exposure of immunogenic mitochondrial components. Use of autologous mitochondria may reduce the risk of immune rejection and donor‐recipient incompatibility, although it does not eliminate risks associated with mitochondrial damage, mtDNA leakage, or poor intracellular routing [3]. Optimized isolation, purification, storage, and delivery protocols are therefore critical to preserve membrane potential, maintain mtDNA integrity, minimize N‐formyl peptide and mtDNA release, and reduce contaminating cellular debris [123]. Overall, mitophagic selection and innate immune sensing jointly determine the post‐transfer fate of donor mitochondria, highlighting the need to balance mitochondrial persistence, quality control, and immune tolerance in future mitoTT applications.

### 3.5 Re‐Establishment of Organelle Contact Sites After mitoTT

Organelle contact sites are critical determinants of mitochondrial function because they coordinate calcium transfer, lipid exchange, redox balance, organelle positioning, and quality control. After mitoTT, transferred mitochondria must not only enter the recipient cell but also become functionally coordinated with the host organelle network. However, direct evidence that transplanted mitochondria re‐establish stable and physiologically productive contacts with the endoplasmic reticulum, lysosomes, or peroxisomes remains limited. Therefore, organelle crosstalk should be discussed as a plausible but incompletely validated checkpoint for post‐transfer mitochondrial persistence and function.

#### 3.5.1 Mitochondria‐Peroxisome Redox and Lipid Coupling

Peroxisomes and mitochondria form a metabolically coupled network that coordinates fatty acid β‐oxidation, lipid metabolism, and ROS regulation [125]. In the post‐transfer setting, peroxisome–mitochondria coupling may help buffer redox imbalance and lipid metabolic stress generated by the uptake or remodeling of donor mitochondria. Peroxisome–mitochondria contact sites have been implicated in lipid exchange, redox signaling, and coordinated organelle dynamics, but whether transferred mitochondria efficiently establish such contacts after mitoTT remains unclear [126, 127]. Thus, peroxisome integrity may influence the redox stability and metabolic adaptation of transferred mitochondria, although this remains an underexplored area in mitoTT research.

#### 3.5.2 Mitochondria‐Lysosome Contacts, Mitophagy, and Endolysosomal Routing

Lysosomes are terminal degradative organelles that remove dysfunctional mitochondria through mitophagy and regulate the fate of endocytosed mitochondrial material [128]. After mitochondrial uptake, lysosomal enzymatic activity and autophagic flux may determine whether internalized mitochondria are degraded, recycled, or escape degradation and persist in the cytosol [129]. Dysfunctional lysosomal activity can also promote secondary mitochondrial impairment and inflammatory signaling, particularly when mtDNA or other mitochondrial damage‐associated molecular patterns accumulate in damaged endolysosomal compartments [130, 131]. Therefore, lysosomal function represents a critical post‐transfer checkpoint that can either eliminate damaged donor mitochondria or limit therapeutic persistence by degrading otherwise functional mitochondrial material. Future studies should distinguish lysosomal degradation from productive cytosolic delivery using live‐cell tracking, lysosomal colocalization assays, endosomal escape markers, and donor‐specific mitochondrial reporters.

#### 3.5.3 Mitochondria‐Endoplasmic Reticulum Contact Sites and MAM Reassembly

The endoplasmic reticulum (ER) forms specialized contact sites with mitochondria known as mitochondria‐associated membranes (MAMs) [61]. MAMs function as communication hubs that regulate calcium transfer, lipid exchange, mitochondrial fission, apoptosis, autophagy, and stress signaling [132]. For transferred mitochondria, productive association with ER contact sites may be important for calcium buffering, lipid remodeling, mitochondrial dynamics, and synchronization with host‐cell stress responses [98]. Conversely, inadequate or abnormal MAM association could impair calcium handling, promote ER stress, increase susceptibility to mitophagy, or limit functional recovery after mitoTT. However, whether donor mitochondria re‐establish stable MAM contacts after transfer has rarely been directly tested and should be assessed using proximity labeling, contact‐site imaging, ER‐mitochondria distance analysis, and donor‐specific mitochondrial tracking.

Collectively, organelle contact‐site biology represents a major unresolved dimension of mitoTT. Future work should determine whether transferred mitochondria merely persist transiently in the cytoplasm or become functionally embedded within ER, lysosomal, and peroxisomal communication networks that regulate calcium handling, lipid metabolism, redox balance, mitophagy, and immune signaling [122, 123].

### 3.6 Integrated Post‐Transfer Quality Control Framework

Post‐transfer mitochondrial quality control should be viewed as an integrated framework rather than a single pathway, because donor mitochondria may be functionally integrated, persist transiently, undergo remodeling, be immunologically sensed, or be degraded upon uptake [3]. In addition to mitophagy, mitochondrial biogenesis pathways regulated by peroxisome proliferator‐activated receptor gamma coactivator 1‐alpha (PGC‐1α), nuclear respiratory factor 1 (NRF1), and mitochondrial transcription factor A (TFAM) may influence whether the recipient cell expands, replaces, or remodels its mitochondrial population after mitoTT. Immune modulation is another component of this framework, because damaged donor mitochondria, fragmented mtDNA, or incompletely degraded mitochondrial cargo may act as mitochondrial damage‐associated molecular patterns (mtDAMPs) [133]. Autologous mitochondrial sources and standardized isolation, purification, storage, and delivery protocols may reduce immunogenicity and improve biocompatibility, but they do not eliminate risks related to mitochondrial damage, poor intracellular routing, or insufficient functional integration [131]. Accordingly, mitoTT efficacy should be assessed using a combination of uptake, persistence, localization, respiratory function, mtDNA maintenance, cristae integrity, organelle contact‐site reassembly, mitophagy, and immune activation readouts. This integrated approach would help distinguish durable mitochondrial integration from short‐term metabolic rescue or indirect paracrine effects.

### 3.7 Determinants of Successful Mitochondrial Integration and Persistence

The success of mitochondrial integration and persistence depends on multiple interrelated variables, including donor mitochondrial viability, donor‐recipient compatibility, mtDNA integrity, mito‐nuclear coordination, recipient‐cell metabolic state, immune sensing, and the balance of mitochondrial dynamics [134] (Figure 3). Together, these parameters determine whether transferred mitochondria are retained, functionally integrated, or eliminated after uptake.

**FIGURE 3:**
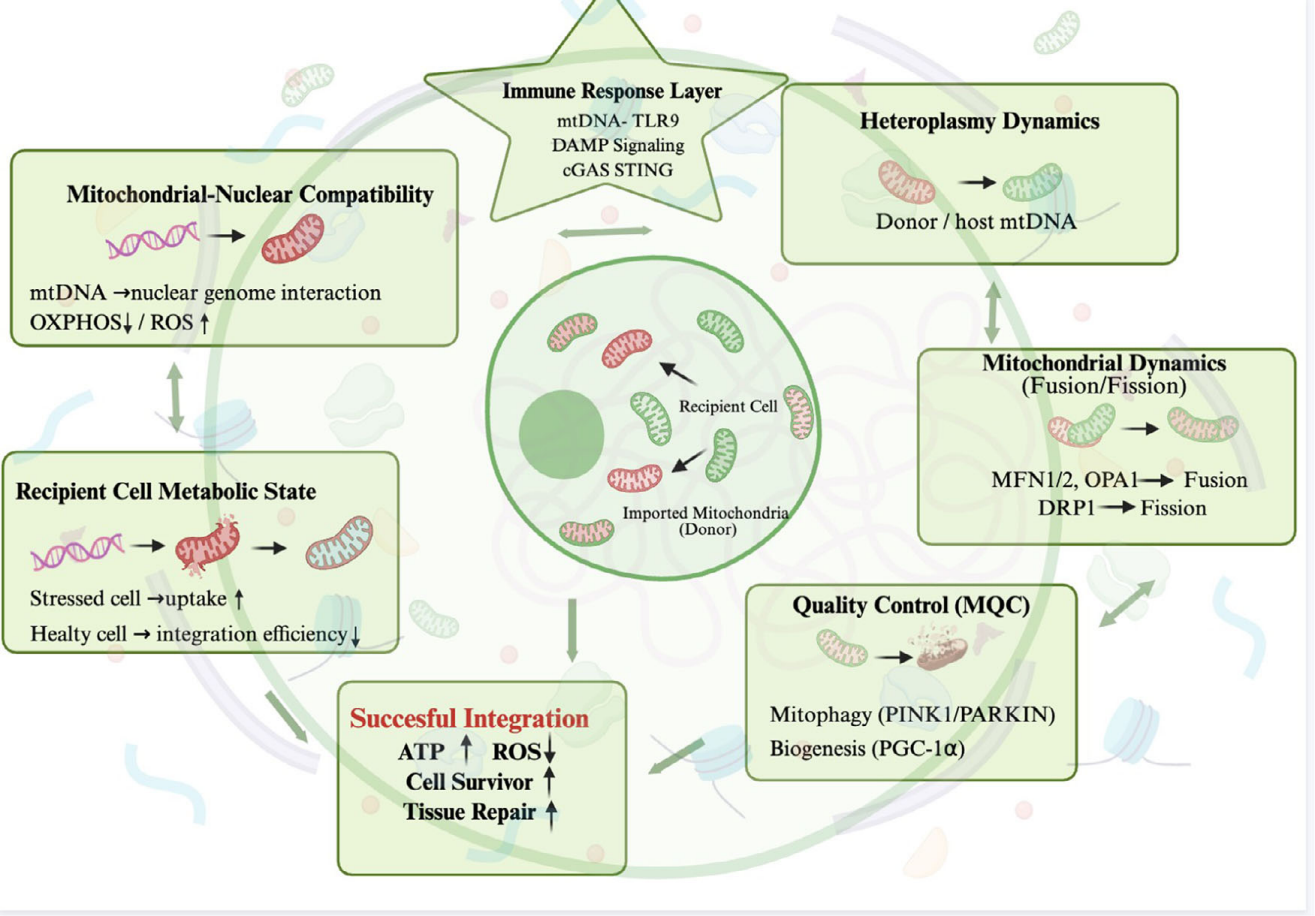
Determinants of successful mitochondrial integration and persistence after mitoTT. The figure summarizes key factors influencing the fate of transferred mitochondria, including mitochondrial‐nuclear compatibility, recipient‐cell metabolic state, immune sensing, donor–host mtDNA heteroplasmy, mitochondrial fusion/fission dynamics, and mitochondrial quality control through mitophagy and biogenesis. Coordinated regulation of these processes may promote successful integration, characterized by increased ATP production, reduced ROS, enhanced cell survival, and tissue repair. Created with BioRender by F. S. Gokdemir (2026).

A central determinant is compatibility between donor mitochondria and the recipient‐cell environment, including bioenergetic demand, redox status, immune context, and availability of nuclear‐encoded mitochondrial proteins [134]. Mitochondria rely on tight coordination between mtDNA‐encoded respiratory‐chain subunits, nuclear‐encoded mitochondrial proteins, and the intracellular metabolic environment [113]. As a result, donor–recipient incompatibility may impair oxidative phosphorylation, disrupt redox balance, increase ROS production, activate mitochondrial quality‐control pathways, and limit donor mitochondrial persistence, even when transferred mitochondria appear structurally intact at the time of delivery [3, 63, 90].

Following transfer, donor and endogenous mitochondrial genomes may coexist within the same recipient cell, potentially generating a heteroplasmic state if donor mtDNA persists [135]. Under these conditions, mtDNA compatibility may influence replication dynamics, respiratory‐chain assembly, mitochondrial quality‐control, and long‐term persistence. Recipient cells may preferentially retain mitochondrial populations that better match local metabolic demands, whereas less compatible or dysfunctional populations may be progressively eliminated through mitophagy or other quality control mechanisms [3, 17, 119]. However, the frequency, stability, and functional consequences of donor‐derived heteroplasmy after mitoTT remain insufficiently defined, particularly in allogeneic or cross‐lineage transplantation settings.

The metabolic state of the recipient cell is another critical determinant of mitochondrial uptake, retention, and functional benefit. Cells experiencing energetic stress, mitochondrial dysfunction, calcium imbalance, or oxidative damage may be more permissive to mitochondrial uptake and may derive greater short‐term benefit from transferred mitochondria [136]. In contrast, cells with sufficient bioenergetic capacity may show reduced dependence on exogenous mitochondria and may clear donor mitochondria more efficiently through quality‐control pathways. This highlights the context‐dependent nature of mitoTT outcomes and reinforces the need to match donor mitochondrial source, delivery route, and recipient disease state [3, 60, 84].

Source selection is therefore a major translational variable. Autologous mitochondria may reduce immune incompatibility and donor‐recipient mismatch, but their quality may be compromised by systemic mitochondrial dysfunction, aging, metabolic disease, or severe inflammation in patients. Allogeneic mitochondria may provide healthier donor organelles, yet they raise additional concerns regarding immunogenicity, mtDNA incompatibility, heteroplasmy, and regulatory standardization [3]. Thus, donor source should be evaluated together with mitochondrial membrane potential, respiratory capacity, mtDNA integrity, sterility, purity, and compatibility with the intended recipient‐cell type.

Overall, successful mitochondrial integration should be defined by a combination of structural, genetic, metabolic, and immunological criteria rather than by uptake alone [137]. Essential evidence should include donor mitochondrial localization, persistence over time, membrane potential, respiratory competence, mtDNA maintenance, cristae integrity, compatibility with the host mitochondrial network, absence of excessive inflammatory activation, and measurable recipient‐cell or tissue‐level functional improvement [3, 13, 62].

Without these criteria, apparent mitoTT benefit may reflect transient metabolic support, paracrine signaling, or selective survival of recipient cells rather than durable donor mitochondrial integration [138].

## 4 Therapeutic Applications and Translational Limitations of mitoTT

Although mitoTT has shown therapeutic potential in multiple preclinical injury models and selected early human applications, its translational status remains experimental. Most available evidence supports short‐term feasibility, local delivery, and acute functional improvement rather than standardized, durable, and broadly generalizable clinical efficacy. Therefore, therapeutic claims should be interpreted according to disease context, delivery route, donor mitochondrial source, biodistribution, persistence, immune response, and the strength of functional validation.

### 4.1 Preclinical Evidence: Acute Functional Rescue Vs. Durable Integration

Preclinical studies indicate that mitoTT can improve functional outcomes in selected models of acute tissue injury, including ischemia–reperfusion injury, skeletal muscle damage, and neural injury [139] (Figure 3). However, many of these outcomes are measured over short experimental windows, and it remains difficult to determine whether the observed benefit reflects durable donor mitochondrial integration, transient bioenergetic support, paracrine signaling, immune modulation, or enhanced survival of recipient cells. In ischemic heart models, mitoTT has been associated with improved cardiomyocyte viability, enhanced oxygen utilization, and reduced tissue damage [140]. For example, mitochondrial delivery from pectoral muscle to porcine myocardium has been reported to improve global cardiac function and limit necrosis and apoptosis. Likewise, in murine myocardial infarction models, MSC‐derived mitochondria have been reported to stimulate angiogenesis and mitigate cellular injury [141]. Collectively, these findings suggest that the preclinical effects of mitoTT are not restricted to a single organ system but are most consistently observed in tissues with high energetic demand or acute mitochondrial dysfunction. Across experimental settings, reported benefits are commonly associated with improved bioenergetic recovery, reduced oxidative damage, and better preservation of tissue architecture [3, 139].

Intramuscular injection of mitochondria has been reported to reduce infarct size, improve muscle function, and accelerate regeneration of necrotic muscle fibers in murine models of hindlimb ischemia [142]. In addition, mitoTT promoted muscle fiber regeneration and improved contractile strength in experimental skeletal muscle injury models. MitoTT has also been reported to protect against doxorubicin‐induced skeletal muscle degeneration, potentially through partial restoration of energy homeostasis [20]. These observations suggest that skeletal muscle is a particularly relevant target for mitoTT, given its high mitochondrial content and dependence on efficient oxidative metabolism. They also support the possibility that mitochondrial supplementation may be beneficial not only in acute ischemic injury but also in chronic or drug‐induced models of muscle injury, although long‐term engraftment, donor mitochondrial turnover, and repeated‐dose safety remain insufficiently defined [20, 142, 143].

In neuronal systems, mitoTT has been investigated as a potential neuroprotective strategy that may support cell survival and functional recovery under conditions of mitochondrial dysfunction [144]. In rat models of spinal cord ischemia, mitoTT has been reported to improve locomotor outcomes and reduce apoptosis, in part through downregulation of inflammatory mediators such as TNF‐α and IL‐6 [145]. This is particularly important in neural tissues, where mitochondrial dysfunction and impaired ATP production can rapidly translate into irreversible functional loss [144, 146]. Similarly, experimental models of traumatic brain injury and Parkinson's disease suggest that mitoTT may reduce neuronal degeneration and microglial activation while supporting ATP production required for synaptic maintenance [147, 148]. Nevertheless, neural applications face additional translational barriers, including delivery across complex neurovascular interfaces, cell‐type‐specific uptake, inflammatory activation, and uncertain long‐term donor mitochondrial persistence.

Overall, preclinical studies support mitoTT as a potentially useful cytoprotective strategy across multiple organ systems, particularly in acute injury contexts. Despite differences in experimental design, target tissue, and delivery route, mitoTT is commonly associated with reduced oxidative stress, improved mitochondrial bioenergetics, and functional recovery in injured tissues [3, 19, 139, 149]. However, several issues remain unresolved, including optimal delivery route, dosing, biodistribution, repeated‐dose safety, immune monitoring, donor mitochondrial persistence, and long‐term integration within recipient cells [3, 150, 151]. Addressing these limitations will be essential for translating encouraging preclinical findings into reliable therapeutic strategies. In this context, early clinical studies should be interpreted primarily as feasibility and safety assessments rather than definitive demonstrations of efficacy.

### 4.2 Clinical Evidence: Early Feasibility Signals and Current Limitations

Early human studies provide preliminary evidence that autologous mitoTT can be technically implemented in selected clinical settings, particularly when mitochondria are delivered locally and within defined procedural windows. However, the current clinical evidence remains limited by small patient numbers, heterogeneous indications, non‐randomized or open‐label designs, limited follow‐up, and lack of standardized outcome measures [152, 153].

The first reported human application involved pediatric patients with ischemia–reperfusion injury undergoing cardiac surgery. In this small cohort, four of five treated patients were weaned from extracorporeal membrane oxygenation (ECMO), and three survived to discharge or follow‐up, indicating potential feasibility in a highly vulnerable patient population. No clear short‐term signal of rejection or severe inflammatory response was reported, although the small sample size limits safety generalization [152]. Despite these limitations, the study provided an early clinical indication that autologous mitoTT could be implemented during cardiac surgical procedures [19, 152].

A subsequent pediatric retrospective study included 24 patients, of whom 10 received mitoTT and 14 served as controls [154]. ECMO separation after one week was reported in 80% of treated patients compared with 29% of controls. These findings support further investigation of mitoTT as an adjunctive strategy in pediatric cardiac ischemia–reperfusion injury and postcardiotomy dysfunction. However, interpretation should remain cautious because the retrospective design, small patient numbers, and limited follow‐up do not allow firm conclusions regarding long‐term efficacy, safety, or generalizability [150, 154].

Beyond cardiac applications, mitochondrial augmentation has also been explored in pediatric patients with single large‐scale mtDNA deletion syndromes using autologous CD34+ hematopoietic cells enriched with maternally derived healthy mitochondria. This compassionate‐use series was reported to be well tolerated in six patients, with decreased heteroplasmy in four patients and increased mtDNA content in all patients. Although some clinical improvement was described, the small cohort size and compassionate‐use design preclude definitive conclusions regarding efficacy.

In chronic pressure wounds, local injection of autologous skeletal muscle‐derived mitochondria has been investigated as a strategy to support tissue repair. Reported outcomes included reduced wound size, increased granulation tissue, and accelerated epithelialization, suggesting potential benefit in local tissue regeneration [153]. However, controlled studies with larger cohorts, longer follow‐up, and standardized wound‐healing endpoints are required before this approach can be considered clinically established.

Autologous mitoTT is also being evaluated in acute ischemic stroke, including intra‐arterial or endovascular delivery during thrombectomy/reperfusion procedures. This represents an early clinical attempt to translate mitoTT into neurological disease, but its interpretation should focus on feasibility, procedural safety, biodistribution, and early outcome signals rather than definitive neuroprotective efficacy [155].

Taken together, the currently available clinical evidence suggests that mitoTT is technically feasible in selected human settings and may provide measurable short‐term benefit, particularly when autologous mitochondria are used and delivery is local, intraoperative, or performed during defined reperfusion windows. However, the number of treated patients remains small, indications are heterogeneous, follow‐up is limited, and standardized criteria for mitochondrial dose, potency, biodistribution, persistence, immune monitoring, and clinical endpoints are not yet established. Therefore, current human studies should be interpreted as early feasibility and safety signals rather than definitive evidence of broad clinical efficacy (Table 2).

**TABLE 2:** Clinical and translational status of mitoTT and mitochondrial augmentation strategies.

Disease / indication	Study type / status	Strategy / delivery	Key outcome or aim	Main limitation	Validation status
Cardiac ischemia–reperfusion injury	Pediatric human study	Autologous skeletal muscle mitochondria; direct myocardial injection	First clinical feasibility report; ECMO weaning and no clear short‐term rejection/inflammation signal reported [152]	Very small cohort; limited follow‐up; uncertain long‐term persistence	Early clinical feasibility
Postcardiotomy refractory cardiogenic shock	Pediatric retrospective study	Autologous mitochondrial transplantation after I/R injury	Higher ECMO separation in treated patients than controls, with improved ventricular recovery [154]	Retrospective design; small cohort; limited generalizability	Retrospective clinical evidence
Single large‐scale mtDNA deletion syndromes	Compassionate‐use pediatric series	Ex vivo mitochondrial augmentation of autologous CD34+ cells	Well tolerated in 6 patients; decreased heteroplasmy in 4/6 and increased mtDNA content reported [156]	Compassionate‐use design; unclear durability	Early clinical / compassionate use
Chronic pressure wound	Human case / early application	Local injection of autologous skeletal muscle mitochondria	Reduced wound size, increased granulation tissue, and accelerated epithelialization reported [153]	Limited cohort; need for standardized wound endpoints	Early clinical feasibility
Acute ischemic stroke	Phase 1 open‐label trial	Intra‐arterial/endovascular autologous mitoTT during reperfusion	First human brain application; feasibility and no major procedural/systemic adverse events reported [157]	Open‐label design; uncertain biodistribution and targeting of brain cells	Phase 1 safety
Ischemic heart disease / ST‐segment elevation myocardial infarction (STEMI)	Prospective randomized trial	Intracoronary autologous platelet‐derived mitochondria	Modest left ventricular ejection fraction (LVEF) improvement and improved exercise capacity reported [158]	Needs larger multicenter validation; dose and persistence unclear	Early randomized evidence
Refractory polymyositis / dermatomyositis	Phase 1/2a trial	Allogeneic umbilical cord MSC‐derived mitochondria, PN‐101	No severe adverse drug reactions; minimal IMACS improvement reported [159]	Allogeneic source; immune safety and mechanism unclear	Phase 1/2a evidence
Pearson syndrome / mitochondrial disease	Registered Phase I/II trial	Mitochondrial augmentation therapy	Designed to evaluate safety and symptom reduction (NCT03384420)	Outcomes pending/registry‐dependent	Registered clinical trial
Refractory polymyositis / dermatomyositis	Registered Phase 2 trial	Allogeneic mitochondria, PN‐101	Follow‐up efficacy and safety trial (NCT07122648)	Efficacy, dosing, and tissue targeting remain to be validated	Registered Phase 2 trial
Low‐risk myelodysplastic syndrome	Registered interventional trial	MNV‐201; autologous CD34+ HSPCs enriched by MAT	Designed to assess safety and preliminary efficacy (NCT06017869)	Manufacturing complexity; engraftment and persistence unclear	Registered interventional trial

***Note***: Validation status was assigned according to the current level of clinical evidence, including reported human use, retrospective or randomized clinical data, early‐phase safety testing, and registered ongoing trial status. None of the listed approaches should yet be considered broadly standardized mitoTT therapy; most remain indication‐specific and require further validation of dose, potency, biodistribution, persistence, immune safety, and long‐term efficacy.

### 4.3 Delivery Barriers and Bioengineering Strategies for mitoTT

Despite encouraging preclinical and early clinical findings, the therapeutic translation of mitoTT remains constrained by major delivery‐related barriers. Isolated mitochondria are biologically active but fragile organelles that may rapidly lose membrane potential, respiratory competence, or structural integrity in extracellular environments. In addition, efficient therapeutic delivery requires mitochondria to reach the target tissue, remain viable during transport, avoid immune recognition, enter recipient cells, escape degradative compartments when necessary, and persist long enough to produce measurable functional benefit. Therefore, delivery strategy is not a technical detail but a central determinant of mitoTT efficacy and safety.

#### 4.3.1 Biological Barriers: Extracellular Instability, Uptake Efficiency, and Immune Recognition

Exposure of mitochondria to the extracellular environment leads to rapid loss of membrane integrity and functional decline [160]. Outside the cell, mitochondria are subjected to a hostile environment characterized by calcium overload, pH fluctuations, oxidative stress, and enzymatic degradation, all of which compromise mitochondrial stability and viability [46, 161, 162]. These conditions accelerate the loss of membrane potential, promote structural deterioration, and increase ROS generation, thereby reducing the likelihood of successful uptake by recipient cells. Consequently, the short lifespan of extracellular mitochondria represents a major limitation for mitoTT [3]. To mitigate these effects, mitochondrial preparations are generally optimized through fresh isolation, controlled cold‐chain handling, and protective buffer systems, although these approaches do not fully prevent functional decline [151].

In addition to extracellular instability, limited cellular uptake remains a major biological barrier for mitoTT. Regardless of whether mitochondria are administered by direct injection, intravascular delivery, or diffusion through tissues, cellular uptake is often limited and may vary substantially according to tissue type, delivery route, mitochondrial preparation, and recipient‐cell state [98]. This limited efficiency reflects multiple biological barriers, including insufficient cell‐surface recognition, restricted endocytic capacity, and rapid extracellular clearance [46, 63, 151].

Mitochondria that are not internalized are susceptible to extracellular damage, phagocytic clearance by macrophages, or dispersal within the tissue matrix [63, 151]. These limitations are further influenced by brief donor–recipient contact time, restricted phagocytosis‐like internalization, and rapid clearance in circulation or extracellular matrices [46, 151]. During systemic administration, mitochondria may lose membrane integrity after exposure to serum proteins and proteases, be cleared by phagocytic cells, or accumulate in filtering organs such as the liver and lungs [2, 163]. In local injections, the extracellular matrix of the target tissue may restrict mitochondrial diffusion and limit contact with recipient cells [46, 164]. Immune activation represents an additional biological barrier. Because mitochondria retain bacterial‐like molecular features, including mtDNA and N‐formyl peptides, damaged or poorly protected transplanted mitochondria may trigger innate immune signaling [165]. This may limit mitochondrial persistence, reduce biocompatibility, and contribute to local or systemic inflammatory responses, particularly when mitochondrial membrane integrity is compromised during isolation, storage, or delivery.

#### 4.3.2 Technical Barriers: Isolation, Storage, Potency, and Scalability

From a technical perspective, the clinical implementation of mitoTT is limited by the lack of standardized protocols for mitochondrial isolation, preservation, and quality assessment [166]. Variations in tissue source, isolation buffers, centrifugation parameters, processing time, and temperature conditions can significantly affect mitochondrial membrane potential, respiratory activity, structural integrity, and overall functional quality [129, 150, 151]. As a result, mitochondria prepared under different laboratory conditions may show substantial variability in viability and therapeutic performance, which complicates reproducibility across studies.

Storage represents another major challenge. Freshly isolated mitochondria are generally preferred, as mitochondrial function declines rapidly over time, even under controlled conditions. Prolonged handling, repeated washing, or inadequate cold‐chain maintenance can reduce ATP production and increase oxidative damage, thereby compromising mitochondrial quality [129, 151, 164]. These limitations complicate the development of practical workflows for clinical use, particularly in acute settings where rapid preparation and immediate administration are required.

Scalability also remains a major challenge. Current mitochondrial isolation procedures are labor‐intensive, time‐sensitive, and highly dependent on operator experience, making them difficult to translate into standardized, large‐scale clinical production [167]. In addition, the absence of universally accepted release criteria for mitochondrial preparations, such as thresholds for membrane potential, oxygen consumption, ATP production, or purity, further limits regulatory readiness and clinical manufacturability [3, 168]. Therefore, resolving these technical barriers will be essential for moving mitoTT from experimental application toward reproducible, regulated, and clinically deployable practice.

#### 4.3.3 Bioengineering Strategies to Improve Stability, Targeting, and Uptake

Several bioengineering strategies are being developed to improve mitochondrial stability, targeting, and uptake efficiency [169]. One major approach involves lipid‐based encapsulation systems, which can shield mitochondria from extracellular stress and help preserve membrane integrity during delivery [170]. By providing a protective interface between the transplanted organelle and the extracellular environment, these systems may prolong mitochondrial survival and improve therapeutic retention [3, 164]. However, lipid encapsulation may also alter mitochondrial bioactivity, affect membrane interactions, increase formulation complexity, and require careful validation of release, uptake, and immune safety.

Another promising strategy is the use of mitochondria‐carrying vesicles and extracellular vesicle‐based delivery systems, including engineered mito‐EVs [171]. These vesicle‐mediated platforms may reduce immune recognition, improve compatibility with recipient cells, and promote intracellular transfer of mitochondrial cargo. In parallel, polymeric carriers, biomaterial‐assisted delivery systems, and surface‐modification approaches are being explored to increase tissue targeting and reduce off‐target loss after administration [3, 25, 98]. However, EV‐based and biomaterial‐assisted platforms remain limited by cargo heterogeneity, isolation reproducibility, scalability, uncertain delivery of intact functional mitochondria, and potential immunogenicity.

In addition, carrier nanoparticles and peptide‐guided targeting systems may help direct mitochondria toward specific tissues or recipient‐cell populations, thereby increasing delivery precision [172]. Although many of these approaches are still at the preclinical stage, they highlight the growing importance of integrating mitochondrial biology with biomaterial design and translational bioengineering [173]. Such strategies may improve the translational feasibility of mitoTT, but their clinical value will depend on standardized validation of mitochondrial viability, potency, biodistribution, persistence, immune safety, and functional benefit [3, 98, 164].

Overall, delivery remains one of the principal translational bottlenecks for mitoTT. Future progress will require matching the mitochondrial source, formulation strategy, delivery route, and disease context with validated potency and safety endpoints. Local autologous delivery may remain most suitable for acute and anatomically accessible injuries, whereas systemic or engineered approaches may be required for diffuse, chronic, or multisystem diseases. However, broader clinical translation will require standardized manufacturing, reproducible dosing, biodistribution tracking, immune monitoring, and long‐term safety assessment.

## 5 Future Directions and Roadmap for Clinical Translation

Future progress in mitoTT will depend on moving from proof‐of‐concept demonstrations toward standardized, mechanism‐informed, and clinically validated therapeutic platforms. The next phase of the field should prioritize not only improved mitochondrial delivery but also validated potency assays, donor–recipient compatibility assessment, donor‐specific tracking, long‐term safety monitoring, and disease‐specific clinical endpoints. These priorities are essential for distinguishing transient metabolic rescue from durable mitochondrial integration and for defining which indications are most likely to benefit from mitoTT.

### 5.1 Standardized Potency, Quality, and Release Criteria

The clinical application of mitoTT requires standardized potency, quality, and release criteria across mitochondrial isolation, purification, storage, and delivery workflows [129]. Current protocols vary substantially in tissue source, isolation method, buffer composition, processing time, storage temperature, mitochondrial purity, membrane potential, ROS production, respiratory competence, and overall biocompatibility [150]. This variability limits reproducibility, complicates comparison across studies, and delays regulatory readiness. Future protocols should therefore define minimum quality thresholds for membrane potential, oxygen consumption rate, ATP production capacity, mtDNA integrity, sterility, endotoxin levels, purity, and dose normalization. Standardized reporting of these parameters would improve cross‐study comparability and support the development of clinically reproducible mitoTT products [174].

### 5.2 Immunological Safety and Donor–Recipient Compatibility

Immunological safety remains a central translational priority for mitoTT. Because mitochondria contain bacterial‐like molecular patterns, including mtDNA and N‐formyl peptides, damaged or poorly controlled mitochondrial preparations may activate innate immune pathways [64, 175]. These pathways may include TLR9, cGAS–STING, inflammasome signaling, and formyl peptide receptor pathways [176]. Future studies should distinguish immune responses caused by mitochondrial damage, donor‐recipient incompatibility, allogeneic recognition, delivery vehicle effects, or disease‐associated inflammation. This distinction will be particularly important when comparing autologous, allogeneic, and engineered mitochondrial sources. Standardized immune‐monitoring panels should include local and systemic cytokine responses, complement activation, leukocyte recruitment, mtDNA release, anti‐mitochondrial responses, and long‐term inflammatory sequelae.

### 5.3 Tracking Persistence, Biodistribution, and Functional Integration

A major unresolved question in mitoTT is how long transferred mitochondria persist, where they are distributed, and whether they become functionally integrated into recipient‐cell networks [146]. Long‐term donor mitochondrial performance depends on maintenance of membrane potential, preservation of mtDNA, respiratory competence, compatibility with the host mitochondrial network, and balanced fusion‐fission dynamics [177]. Donor mitochondria that fail to adapt to the recipient cell's intracellular environment may be eliminated through mitophagy, degraded in lysosomes, or trigger immune sensing if mitochondrial damage‐associated molecules are exposed [178]. Therefore, strategies aimed at prolonging mitochondrial lifespan should be evaluated not only by short‐term ATP production or membrane potential but also by donor‐specific persistence and functional contribution over time [120]. Future studies should combine donor‐specific mitochondrial tracking with functional assays to determine whether transferred mitochondria remain intact, maintain mtDNA, preserve cristae structure, contribute to respiration, re‐establish organelle contact sites, or undergo mitophagic degradation [3]. Useful approaches may include donor‐specific mtDNA variant tracing, live‐cell imaging, mitochondrial membrane potential assays, single‐cell biodistribution analysis, respiratory flux measurements, electron microscopy, and long‐term in vivo tracking. These methods are necessary to distinguish durable mitochondrial integration from transient metabolic support, indirect paracrine effects, or selective survival of recipient cells.

### 5.4 Trial Design and Clinical Endpoints

Future clinical translation will require trial designs that match mitoTT strategy to disease context, delivery route, donor mitochondrial source, and expected mechanism of benefit [179]. Acute, anatomically accessible injuries may be more suitable for local autologous delivery, whereas chronic, diffuse, or multisystem diseases may require engineered, systemic, or cell‐based mitochondrial augmentation platforms. Clinical trials should move beyond feasibility endpoints alone and include predefined measures of safety, dose, biodistribution, mitochondrial persistence, immune activation, organ‐specific functional recovery, and long‐term follow‐up [168]. Regulatory readiness will also require scalable manufacturing, good manufacturing practice (GMP)‐compatible processing, validated release testing, sterility controls, batch consistency, and traceable dose metrics [180]. Without these criteria, mitoTT will remain difficult to compare across studies and challenging to translate into standardized clinical practice.

### 5.5 Integrated Roadmap for Next‐Generation mitoTT

Next‐generation mitoTT should be developed as an integrated therapeutic platform rather than as isolated organelle delivery alone [3]. This roadmap should combine optimized donor selection, mitochondrial quality control, protective formulation, targeted delivery, donor‐specific tracking, immune safety monitoring, and disease‐specific efficacy endpoints [181]. Bioengineering approaches, including lipid encapsulation, extracellular vesicle‐based carriers, biomaterial‐assisted local delivery, surface modification, and peptide‐guided targeting, may improve mitochondrial stability and targeting [182, 183]. However, their value will depend on rigorous validation of mitochondrial viability, potency, biodistribution, persistence, immune safety, and functional benefit. For systemic applications, targeted delivery strategies should also be evaluated for off‐target accumulation, vascular trapping, uptake specificity, and inflammatory activation. Ultimately, the future of mitoTT will depend on integrating mitochondrial biology with biomaterial science, nanotechnology, targeted delivery design, and clinically meaningful validation endpoints [119, 129]. The field should prioritize indications in which the mechanism of benefit is biologically plausible, delivery is technically feasible, and clinical outcomes can be measured within meaningful follow‐up windows.

## 6 Conclusion

MitoTT represents a rapidly evolving therapeutic concept at the intersection of cell biology, bioenergetics, immunology, and regenerative medicine. Current evidence indicates that mitochondria can be transferred through multiple endogenous and experimental routes, including TNTs, EV‐associated pathways, cell fusion, gap junction‐associated communication, and phagocytosis‐like internalization. However, these routes differ substantially in cargo type, directionality, efficiency, biological range, and therapeutic interpretability. Therefore, mitoTT should not be viewed simply as mitochondrial delivery but as a multi‐step biological process involving uptake, intracellular routing, network interaction, cristae remodeling, mtDNA maintenance, organelle contact‐site reassembly, immune sensing, and quality‐control.

Preclinical studies support the potential of mitoTT to improve bioenergetic recovery and tissue repair in selected injury models, particularly in organs with high metabolic demand. Early clinical studies further suggest that autologous mitochondrial transplantation or mitochondrial augmentation can be technically feasible in defined clinical settings. Nevertheless, the field remains at an early translational stage. Small patient numbers, heterogeneous indications, limited follow‐up, nonstandardized mitochondrial preparations, uncertain biodistribution, incomplete donor‐specific tracking, and unresolved immunological risks currently prevent broad clinical conclusions.

Future progress will depend on the development of standardized potency assays, reproducible manufacturing workflows, validated delivery strategies, donor–recipient compatibility assessment, immune safety monitoring, and long‐term tracking of mitochondrial persistence and function. In particular, distinguishing durable mitochondrial integration from transient metabolic rescue, paracrine effects, or selective survival of recipient cells will be essential. With rigorous mechanistic validation and carefully designed clinical studies, mitoTT may evolve from an experimental rescue approach into a more precise and clinically actionable mitochondrial therapeutic platform.

## Author Contributions

**F. Seyma Gokdemir**: conceptualization, investigation, writing – original draft, writing – review and editing. **Yagmur Ozendi**: investigation, writing – original draft, writing – review and editing. **Gokhan Burcin Kubat**: conceptualization, writing – review and editing. **Keshav K. Singh**: conceptualization, writing – review and editing.

## Conflicts of Interest

Keshav K. Singh is a co‐founder of and holds equity in Yuva Biosciences and serves on its Scientific Advisory Board. The other authors declare no competing interests.

## Data Availability

Data sharing does not apply to this article as no new data were created or analyzed in this study.
